# Posthumous dignity therapy: Challenges and opportunities in the Brazilian cultural context

**DOI:** 10.1017/S1478951525000276

**Published:** 2025-04-07

**Authors:** Ana Carolina Kotinda Bennemann, Carlos Eduardo Paiva, Bianca Sakamoto Ribeiro Paiva

**Affiliations:** 1 Palliative Care Unit, Londrina’s Cancer Hospital, Londrina, Brazil; 2 GPQual - Research Group on Palliative Care and Quality of Life - Barretos Cancer Hospital, Barretos, Brazil; 3 Teaching and Research Institute, Barretos Cancer Hospital, Barretos, Brazil; 4 Department of Clinical Oncology, Breast and Gynecology Division, Barretos Cancer Hospital, Barretos, Brazil

When faced with a life-threatening illness, we discover the wisdom and peace that the thought of mortality may bring. We can reflect on our past, acknowledge our unique qualities, and contemplate our proudest accomplishments. What if we put all these thoughts on paper, creating a legacy to share our story and solace to our loved ones with our words, even after we are gone?

Dignity Therapy (DT) is a brief psychotherapy aimed at alleviating emotional suffering, restoring dignity, and affirming patients’ identity beyond their illness (Chochinov et al. 2005). It uses a semi-structured interview to explore life stories and desired legacies. The recorded interview is transcribed, edited, and shared as a legacy document for loved ones. As a response to the inability of some patients to participate in DT during their lifetime due to the severity of their illness, cognitive decline, or the unavailability of trained therapists, Posthumous Dignity Therapy (PDT) was developed. Created by (Julião et al. 2023), PDT addresses this gap by offering a brief psychotherapeutic approach conducted on the family members of deceased patients, aiming to aid the family during their grieving process (Julião et al. 2023), enabling them to feel connected to the departed and providing a tangible way to honor and safeguard the memory of their significant others (Bennemann et al. 2024).

Narrowing to the Brazilian cultural context, we emphasize that several factors, including religious diversity, social inequalities, and the difficulty of openly discussing death, create additional barriers to implementing this approach (Bennemann et al. 2024). Moreover, the stigma surrounding emotional suffering for both patients and their families may hinder the acceptance and effectiveness of PDT. This underscores the importance of adaptations that consider Brazil’s sociocultural specificities.

Although death is a natural and biological event that is part of the entire life cycle, the process of dying must be understood within a social and cultural context, as it is influenced by time and space. In Brazilian culture, death and dying are sensitive topics, and conversations about them often face barriers. Supporting an article by Valentino et al (Valentino et al. 2023) that highlights the lack of death education in the Brazilian population, the study on the validation and cross-cultural adaptation of the PDT Schedule of Questions to brazilian portuguese (Bennemann et al. 2024) revealed that some participants expressed discomfort with the subject. The term “posthumous” triggered strong emotional reactions, inducing fear or distress in approximately 30% of the participants. One of the study participants stated that he would not take part in the study because he believed that “discussing someone who had passed away could bring death closer to him or his family members.” This demonstrates the deep-seated bias and hostility still existing in Brazilian culture toward subjects related to death and dying emphasizing the need for cultural validation when talking about patients who are nearing death or have passed away (Paiva et al. 2023).

Educating people about the end-of-life requires a multi-faceted approach that integrates psychological, social, and cultural perspectives. Death education in schools can help normalize and reduce the stigma surrounding this topic. It can be tailored to different ages and levels of understanding, enabling students to better cope with death and other losses (Friesen 2020). In an educational continuum, it is important to incorporate community-based death preparation into individuals’ everyday lives, as people can face losses unexpectedly. Workshops for adults and caregivers can cover key topics such as advanced care planning, hospice care, and grief management. These programs equip participants with practical tools to navigate the dying process and support their loved ones. Additionally, they offer various benefits, including reducing death-related anxiety and stress, fostering a positive awareness of death, and encouraging individuals to appreciate their lives (Park 2022). During our research on Posthumous Dignity Therapy (PDT), we noticed a significant need for more personalized support for participants, both before and after they answered research questions. It became evident that they required someone to listen to their concerns and to guide them through issues related to the dying process, as well as the emotional distress that could arise from discussing their deceased loved ones. This observation highlights the need for death education in Brazilian society.

Apart from the cultural construction of the taboo, gaps in the training of healthcare workers result in the perpetuation of common assumptions in teaching and learning scenarios, which hinders the development of an environment that could effectively support their training in discussing topics related to death and dying during their undergraduate courses (Silva Filho and Minayo 2021). Engaging in discussions about these topics is essential for professionals, as it helps them develop their perspectives and personal questions. These discussions also serve as a foundation for enhancing communication skills with patients and their families. Becoming a dignity therapist requires exceptional interpersonal skills, self-awareness, and an open mind to listen to, validate, and address various sensitive topics related to a person’s life story. A lack of ability to communicate with empathy and assertiveness can hamper open discussions about death and dying, making it challenging to connect with patients and families. This, in turn, can lead to increased psychological distress among professionals. Professionals can build rapport with patients and their caregivers by fostering these discussions.

Rapport is the bond of trust and respect built between the therapist and the research subject. It is particularly important in PDT studies, as it facilitates the research process and enhances the quality and quantity of the data collected while also ensuring the comfort and well-being of participants and researchers alike. The deep-rooted taboos within Brazilian culture regarding this topic, combined with insufficient training for health workers, hinder the establishment of connections, leading to greater challenges for researching these subjects in the country.

Brazil is a developing country that faces significant challenges in studying death and the end-of-life process, largely due to a strong cultural aversion to discussing these topics. Despite these challenges, Brazil has made progress through various initiatives aimed at addressing the issue.

In recent years, there has been a gradual increase in the inclusion of palliative care courses in health program curricula. Since 2022, these courses have become a requirement in medical education. Besides these initiatives, the government has been actively working to enhance access to palliative care for the population. This includes the publication of resolutions that organize palliative care within the Unified Health System (Sistema Único de Saúde – SUS) and the establishment of a National Palliative Care Policy within the SUS. These efforts mark a significant milestone in strengthening palliative care in Brazil. Additionally, there has been a rise in movements aimed at enhancing health professionals’ awareness of self-knowledge and self-care. These movements are essential for helping professionals manage daily challenges in healthcare and discussions about death and dying.

We believe these strategies will gradually help create a new culture around end-of-life topics, encouraging open discussions about death and dying in social settings. As a result, the general population will be better equipped to understand and address these issues, integrating them more into everyday life.

Given the ongoing research in palliative care, especially regarding PDT, a significant increase in both the quantity and quality of information produced is anticipated, considering the cultural transition currently taking place in Brazil. In our clinical practice applying PDT, we have observed that family members express satisfaction and gratitude when being listened to and comforted during their time of mourning. They also highly value the legacy document produced through the therapy.

Enhancing palliative care skills equips healthcare professionals to effectively address the emotional and psychological needs of grieving families. Additionally, as the public becomes increasingly open to discussing issues surrounding death and dying, it fosters the acceptance and implementation of PDT. This progress promotes a more holistic and compassionate approach to support bereaved family members. We believe that both PDT and other methods for addressing emotional pain related to grief hold significant potential for effective use in daily clinical practice, especially within palliative care and other contexts involving the loss of loved ones.

**Figure 1.**
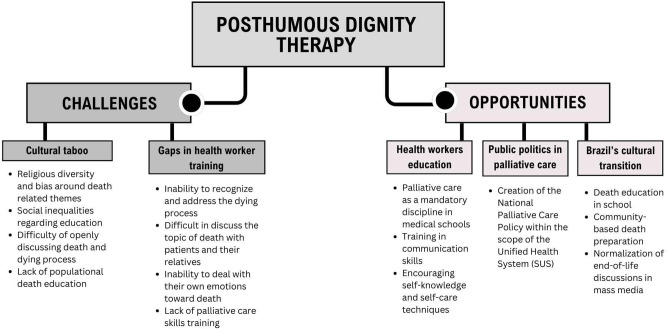
Conceptual map of the challenges and opportunities of Posthumous Dignity Therapy in the Brazilian cultural context.

Challenges and opportunities of Posthumous Dignity Therapy in the Brazilian cultural context can be summarized in Figure 1.
